# Associations Between Incident Asthma With Comorbidity Profiles, Night Sleep Duration, and Napping Duration Trajectories: A 7-Year Prospective Study

**DOI:** 10.3389/ijph.2022.1604939

**Published:** 2022-07-01

**Authors:** Zhigang Hu, Yufeng Tian, Xinyu Song, Ke Hu, Ailan Yang

**Affiliations:** ^1^ Department of Respiratory and Critical Care Medicine, The First College of Clinical Medicine Science, China Three Gorges University, Yichang, China; ^2^ Department of Respiratory and Critical Care Medicine, Yichang Central People’s Hospital at Zhijiang, Zhijiang, China; ^3^ Department of Respiratory and Critical Care Medicine, Yichang Central People’s Hospital, Yichang, China; ^4^ Department of Academic Management, China Three Gorges University, Yichang, China; ^5^ Department of Respiratory and Critical Care Medicine, Renmin Hospital of Wuhan University, Wuhan, China

**Keywords:** incidence, comorbidity, asthma, napping, sleep duration

## Abstract

**Objectives:** We aim to determine whether comorbidity profiles, night sleep duration, and napping duration trajectories were associated with incident asthma in Chinese adults.

**Methods:** A total of 7,655 community-dwelling individuals were included in this study. Latent class/profile analysis(LCA/LPA) identified comorbidity profiles, night sleep duration, and napping duration trajectories. A generalized additive model with binomial regression assessed the associations between incident asthma with sleep trajectories.

**Results:** During a 7-year follow-up period, 205 individuals were newly diagnosed with asthma. LPA identified four trajectories of night sleep duration: dominant short (*n* = 2,480), dominant healthy-long (*n* = 1,405), long decreasing (*n* = 1875), and short increasing (*n* = 1895). We also found three trajectories of napping duration: short increasing (*n* = 3,746), stable normal (*n* = 1,379), and long decreasing (*n* = 2,530). We found three comorbidity profiles: dominant heart diseases or risks (*n* = 766), multiple disorders (*n* = 758), and minimal or least disorders (*n* = 6,131). Compared with dominant short night sleep duration, three other trajectories were associated with significantly decreasing incident asthma. Minimal or least disorders profile was associated with a significant reduction of new-onset asthma than two other comorbidity profiles in dominant short night sleep duration.

**Conclusion:** Our findings suggested that a dominant short night sleep duration trajectory potentially increases incident asthma in Chinese adults.

## Introduction

Asthma is an important public health problem with high incidence and prevalence worldwide that is a huge burden on the economy as well as the psychology of patients. Asthma-attributable deaths and disability-adjusted life-years rates were 6.48 and 297.92 per 100,000 persons in the Global Burden of Disease Study 2017, respectively [1]. A recent study demonstrated that American adolescents and adults with uncontrolled asthma are estimated to be associated with a total economic burden of $963.5 billion and 15.46 million quality-adjusted life years lost over the next 20 years [2]. Therefore, identifying independent factors for developing asthma is a public health priority in order to reduce the substantial burden (secondary to asthma itself) this has on patients and society.

The occurrence of asthma is related to the following risk factors: certain demographics, developmental, lifestyle, virus infection, medication, diet, and inhaled exposures [3]. Depression has a bidirectional association with asthma but also affects asthma-related exacerbation and death [4, 5]. Obesity, which may be classified as central and general obesity, has been identified as an important risk factor for the occurrence and development of asthma. Compared with general obesity, central obesity was more available to reflect body fat distribution and have a greater effect on comorbidity and death [6]. Comorbidity profiles potentially affect asthma severity and asthma control levels and brings increased attention to asthma management [7]. The cross-sectional studies indicated that short sleep duration may increase the prevalence of asthma [8], the frequency of asthma-related emergency department visits [9], and lung function impairment [10]. However, only one prospective study has explored the effect of different sleep duration trajectories from adolescence into adulthood on incident asthma in young adults aged 24–32 years [11]. Whether sleep duration trajectories have different influences in specific populations remains to be studied.

Short sleep duration is likely to augment the risk of developing asthma via multiple mechanisms [8, 11, 12]. Short sleep duration in a specific population, such as people who are overweight/obese, may have a more significant effect on the prevalence of asthma compared with the general population [13]. Therefore, this study aims to determine the associations between incident asthma with night sleep duration and napping duration trajectories through a large 7-year prospective study in the Chinese population. In addition, we investigate whether different sleep duration trajectories influence the incident risk in specific populations (comorbidity profiles, depression, and central obesity).

## Methods

### Study Population

The China Health and Retirement Longitudinal Study (CHARLS), established between June 2011 and March 2012, is a nationally representative longitudinal study for Chinese community residents aged 45 years or older that covers 150 county-level units, 450 village-level units, and 17,708 individuals in 28 provinces.^14^ The CHARLS aims to collect high-quality datasets to assess the associations between social, economic, and health status in middle-aged and older populations. These samples were followed every 2–3 years in the form of personal computer-assisted face-to-face interviews. In wave 1, a total of 13,978 individuals (78.9%) received physical performance and anthropometric measures. Until 2018, individuals have received three other waves of follow-up data collection: wave 2 in 2013, wave 3 in 2015, and wave 4 in 2018. New individuals were added to each wave of the CHARLS. The Biomedical Ethics Review Committee of Peking University approved the CHARLS. The National School of Development at Peking University collected written informed consent from all individuals. A more detailed description of the CHARLS has been reported elsewhere [14].

### Assessment of Night Sleep Duration and Napping Duration

Data on night sleep duration and napping duration came from two questionnaires: “During the past month, how many hours of actual sleep did you get at night?” and “During the past month, how long did you take a nap after lunch?”

For adults, 7–8 h were regarded as an adequate duration for a healthy night’s sleep. Like previous studies, night sleep duration was divided into five groups: <6 h means a very short sleep duration; 6–6.99 h means a short sleep duration; 7–7.99 h means a healthy sleep duration; 8–8.99 h means a long sleep duration; and ≥9 h means a very long sleep duration [15].

In the Chinese population, a napping duration ≥60 min was associated with an increased risk of metabolic syndrome [16] and hypertension [17] compared with no nap. In this study, napping duration was categorized into four groups: 0 min means no nap; 0–59 min means a healthy napping duration; 60–119 means a long napping duration; and ≥120 min means a very long napping duration.

### Assessment of Asthma, Comorbidity, Depression, and Central Obesity

In wave 1 of the CHARLS, the diagnosis of asthma was based on a positive answer to the following question: “Have you ever been diagnosed with asthma by a doctor?“. A total of 637 individuals with doctor-diagnosed asthma were excluded from our study. In three other waves, new-onset asthma depended on positive answers to two more questions: “Have you been diagnosed with asthma by a doctor since the last interview?” and “How did you know that you had asthma? Through routine or a CHARLS physical examination or any other method?”. With the exception of asthma, the CHARLS also obtained data on 13 other physician-diagnosed chronic illnesses, including hypertension, dyslipidemia, hyperglycemia, cancers, chronic lung diseases, liver diseases, heart diseases, stroke, kidney diseases, digestive diseases, emotional or psychiatric problems, memory-related diseases, and arthritis or rheumatism.

The 10-item Center for Epidemiological Studies–Depression Scale (CES-D10) was used to evaluate whether individuals exist depression in the CHARLS. CES-D10 included 10 items on the depressive feelings and behaviors of individuals over 1 week, and every item provided four answers. Scores of every item ranged from 0 to 3; thus, the total score of the CES-D10 fluctuated between 0 and 30. Similar to previous studies, CES-D10 ≥ 12 scores were considered depression [18–20]. Central obesity was measured by the waist to height ratio (WHtR) > 0.5 [21].

### Variables

The following variables were included in this study as potential confounding factors: sex (male vs. female), age (<65 years vs. ≥ 65 years), region (North vs. East and Central vs. West), urban/rural (urban vs. rural), education levels (low vs. middle vs. high), married status (current unmarried vs. current married), smoking (never vs. ever vs. current), alcohol (never vs. ever vs. current), body mass index (BMI), and muscle strength.

No formal education and education below that of primary school was classified as a low education level. Middle education levels included the following education: home school, elementary school, middle school, and high school. Vocational school and colleges or above were categorized as a high education level. According to recommendation by the WHO, BMI was divided into the following four groups: underweight (<18.5 kg/m^2^), normal (18.5 to <24.0 kg/m^2^), overweight (24.0 to <28.0 kg/m^2^), and obesity (≥28.0 kg/m^2^). ^21^ Handgrip strength <28.0 kg for men and <18.0 kg for women were deemed as low skeletal strength [22].

### Statistical Analysis

This study mainly included four components. First, latent class and profile analyses were used to identify the profiles of 13 physician-diagnosed comorbidities, and the trajectories of night sleep duration and napping duration, respectively. The Akaike information criterion, Bayesian information criterion, sample-size adjusted Bayesian information criterion, entropy values, and Vuong-Lo-Mendell-Rubin likelihood ratio test were used to determine the best fitting latent model and class [7, 23]. Subsequently, SPSS compared the characteristics of individuals grouped by night sleep duration trajectories. Categorical variables were presented through counts and percentages (%) and compared the difference among different groups via a chi-square test. Means and standard deviations described continuous variables with the Mann-Whitney U test for skewed continuous variables and Student’s t-test or one-way ANOVA for normally distributed continuous variables. Second, three models assessed the associations between the incidence of new-onset asthma with night sleep duration and napping duration trajectories by using generalized additive analysis with binomial regression. The first model adjusted sex, age, region, urban/rural, education levels, and married status. Model 2 included the following variables: sex, age, region, urban/rural, education levels, married status, smoking, alcohol, body mass index, and low skeletal strength. Model 3 added to the adjustment of depression, central obesity, and comorbidity profiles on the basis of model 2. In addition, we also estimated the correlation between night sleep duration and incident asthma by model 3 with the adjustment of napping duration trajectories. Third, this study respectively explored whether sleep duration trajectories bring different results for specific populations (depression, central obesity, and comorbidity profiles). Four, the study estimated the mediation effect of depression, central obesity, and comorbidity profiles in the association between sleep duration trajectories and incident asthma via mediation analysis. Mediation analysis can provide the total effect, average direct effect, average mediation effect (indirect effect), and average mediated proportion of a mediation factor [24]. SPSS, Empower® (www.empowerstats.com; X&Y solutions, Inc. Boston MA), and Mplus completed all analyses. Odds ratios (ORs) with 95% confidence intervals (CIs) represented the strength of association; meanwhile, statistical significance was defined by a two-tailed *p* < 0.05.

## Results

A total of 7,655 community-dwelling individuals without asthma from the CHARLS 2011 completed four follow-up waves and provided detailed information on all included variables. During the 7-year follow-up period, 205 (2.7%) individuals were diagnosed with new-onset asthma. The mean age was 58.45 ± 13.45 years with 24.6% being of the elderly population (≥65 years) and 52.9% being female. A high proportion of individuals were currently married (89.1%) and lived in rural ares (62.2%). Individuals who currently smoked or ingested alcohol made up 30.8% and 24.3% of the study population, respectively. Individuals who were overweight or showed general obesity made up 40.9%. Mean napping duration gradually rose from 30 ± 41 min in 2011 to 41.5 ± 51 min in 2018, whereas mean night sleep duration slightly decreased from 6.4 h to 6.2 h with an increasing proportion of short and very short night sleep duration. Around 33.7% of individuals were diagnosed with depression and 69.8% with central obesity. In the CHARLS 2011, individuals with new-onset asthma were associated with significantly higher mean age and proportion of very short night sleep duration, depression, diabetes, chronic lung diseases, heart diseases, and arthritis or rheumatism compared with those without new-onset asthma.

**FIGURE 1:**
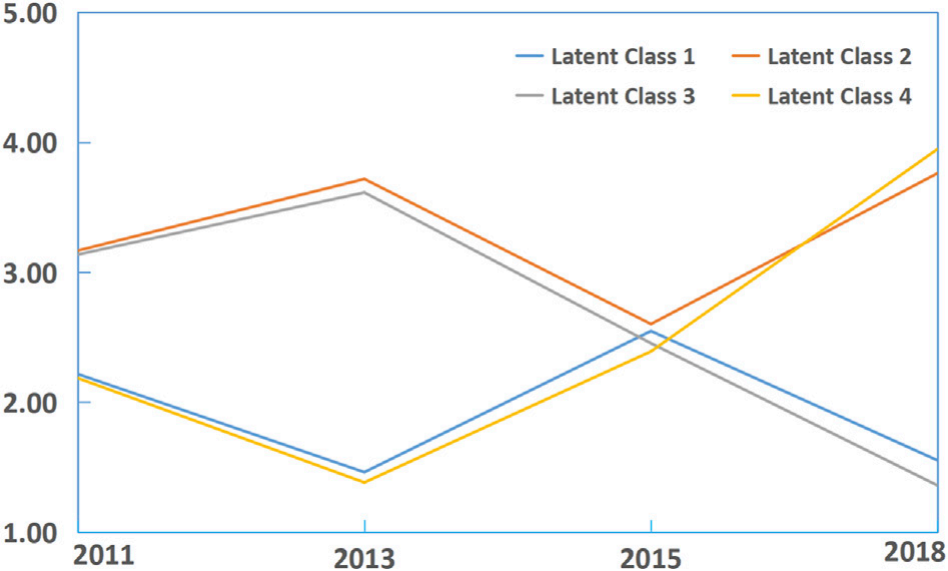
Latent class analyses of night sleep duration trajectories in individuals without asthma from the China Health and Retirement Longitudinal Study 2011. class 1, dominant short (reference, *n* = 2,480, 32.4%); class 2, dominant healthy-long (*n* = 1,405, 18.4%); class 3, long decreasing (*n* = 1875, 24.5%); class 4, short increasing (*n* = 1895, 24.8%). Night sleep duration was divided into five groups:(1) <6 h means a very short sleep duration; (2) 6–6.99 h means a short sleep duration; (3) 7–7.99 h means a healthy sleep duration; (4) 8–8.99 h means a long sleep duration; (5) ≥ 9 h means a very long sleep duration (China. 2022).

**FIGURE 2:**
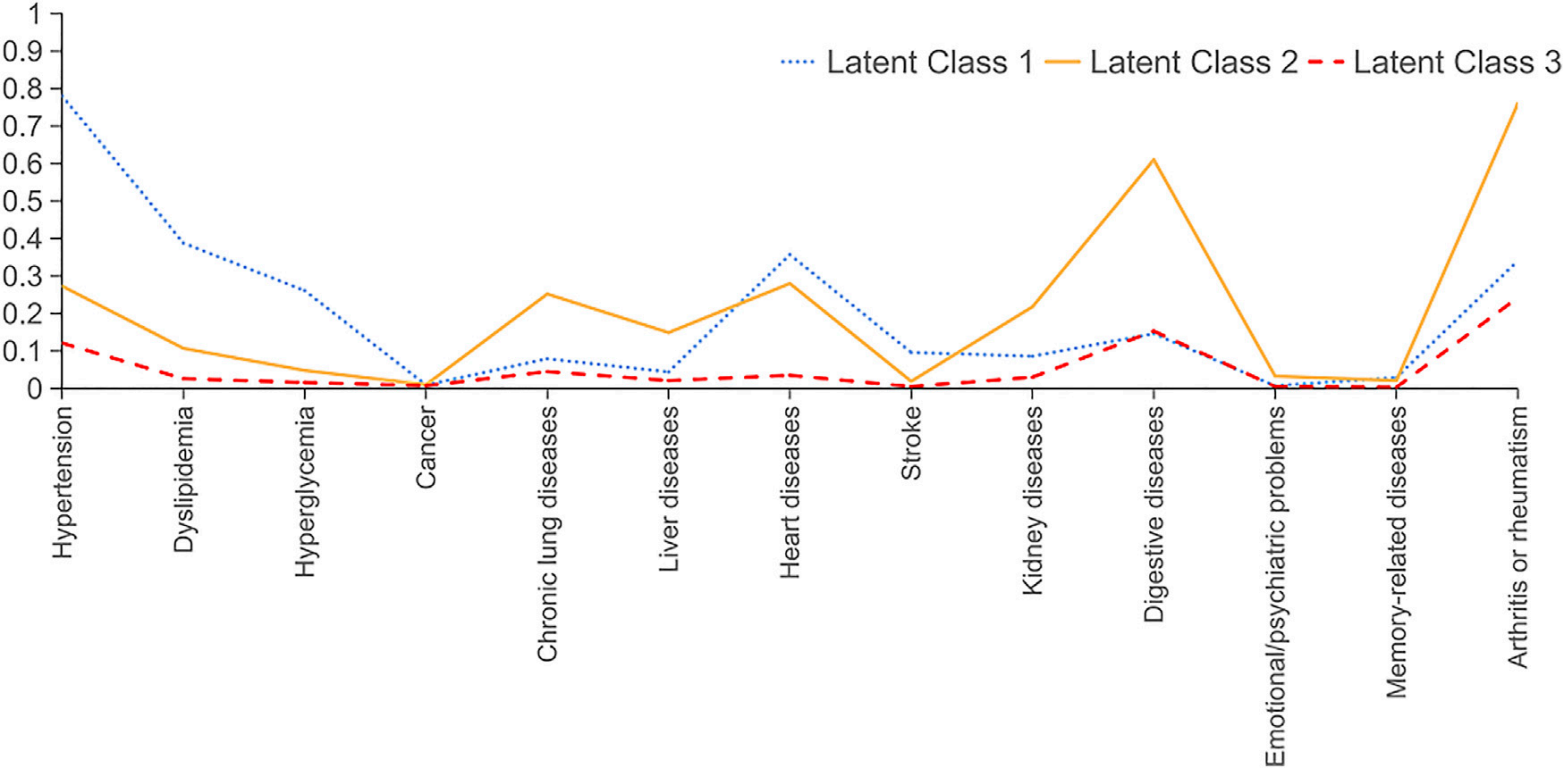
Latent class analyses of comorbidity profiles trajectories in individuals without asthma from the China Health and Retirement Longitudinal Study 2011: class 1, dominant heart diseases or risks (reference, *n* = 766, 10%); class 2, multiple disorders (*n* = 758, 9.9%); class 3, minimal or least disorders (*n* = 6,131, 80.1%) (China. 2022).

Regarding night sleep duration, the best fit latent profile analysis model (see Supplementary Table S1) delineated four trajectory groups (see Figure 1): dominant short (*n* = 2,480, 32.4%, latent Class 1), dominant healthy-long (*n* = 1,405, 18.4%, latent Class 2); long decreasing (*n* = 1875, 24.5%, latent Class 3); and short increasing (*n* = 1895, 24.8%, latent Class 4). Mean night sleep duration <6 h existed in three of four waves in group 1, while a high proportion of mean night sleep duration >7 h was shown in group 2. From the beginning of wave 2, there was gradually decreasing mean night sleep duration in group 3 and increasing mean night sleep duration in group 4. We also identified three trajectories of napping duration (see Supplementary Figure S1; Supplementary Table S1) and three comorbidity profiles (see Figure 2; Table 1) as the best fit model. Three trajectories were named short increasing (*n* = 3,746, 48.9%, latent Class 1), stable normal (n = 1,379, 18%, latent Class 2), and long decreasing (*n* = 2,530, 33.1%, latent Class 3). Three comorbidity profiles were labeled as dominant heart diseases or risks (*n* = 766, 10%, latent Class 1), multiple disorders (*n* = 758, 9.9%, latent Class 1), and minimal or least disorders (*n* = 6,131, 80.1%, latent Class 1). Dominant heart diseases or risks profile was characterized by high probabilities for heart diseases with related risk factors (hypertension, dyslipidemia, and hyperglycemia). A multiple disorders profile was characterized by a combination of the following disorders: high probabilities for hypertension, chronic lung diseases, heart diseases, digestive diseases, and arthritis or rheumatism. A minimal or least disorders profile was characterized by the lowest probability for 13 physician-diagnosed chronic illnesses.

**TABLE 1:** Probability of each indicator variable across three profiles of comorbidities in individuals without asthma from the China Health and Retirement Longitudinal Study.

	Latent class 1	Latent class 2	Latent class 3
	Dominant heart	Multiple	Minimal or
	diseases or risks	disorders	least disorders
	(*N* = 766)	(*N* = 758)	(*N* = 6,131)
Hypertension	0.78	0.273	0.121
Dyslipidemia	0.388	0.107	0.026
Hyperglycemia	0.261	0.048	0.016
Cancer	0.009	0.01	0.007
Chronic lung diseases	0.079	0.252	0.045
Liver diseases	0.044	0.149	0.021
Heart diseases	0.357	0.28	0.035
Stroke	0.096	0.019	0.005
Kidney diseases	0.086	0.218	0.03
Digestive diseases	0.146	0.61	0.153
Emotional/psychiatric problems	0.007	0.033	0.005
Memory-related diseases	0.029	0.021	0.003
Arthritis or rheumatism	0.34	0.759	0.242

**FIGURE 3:**
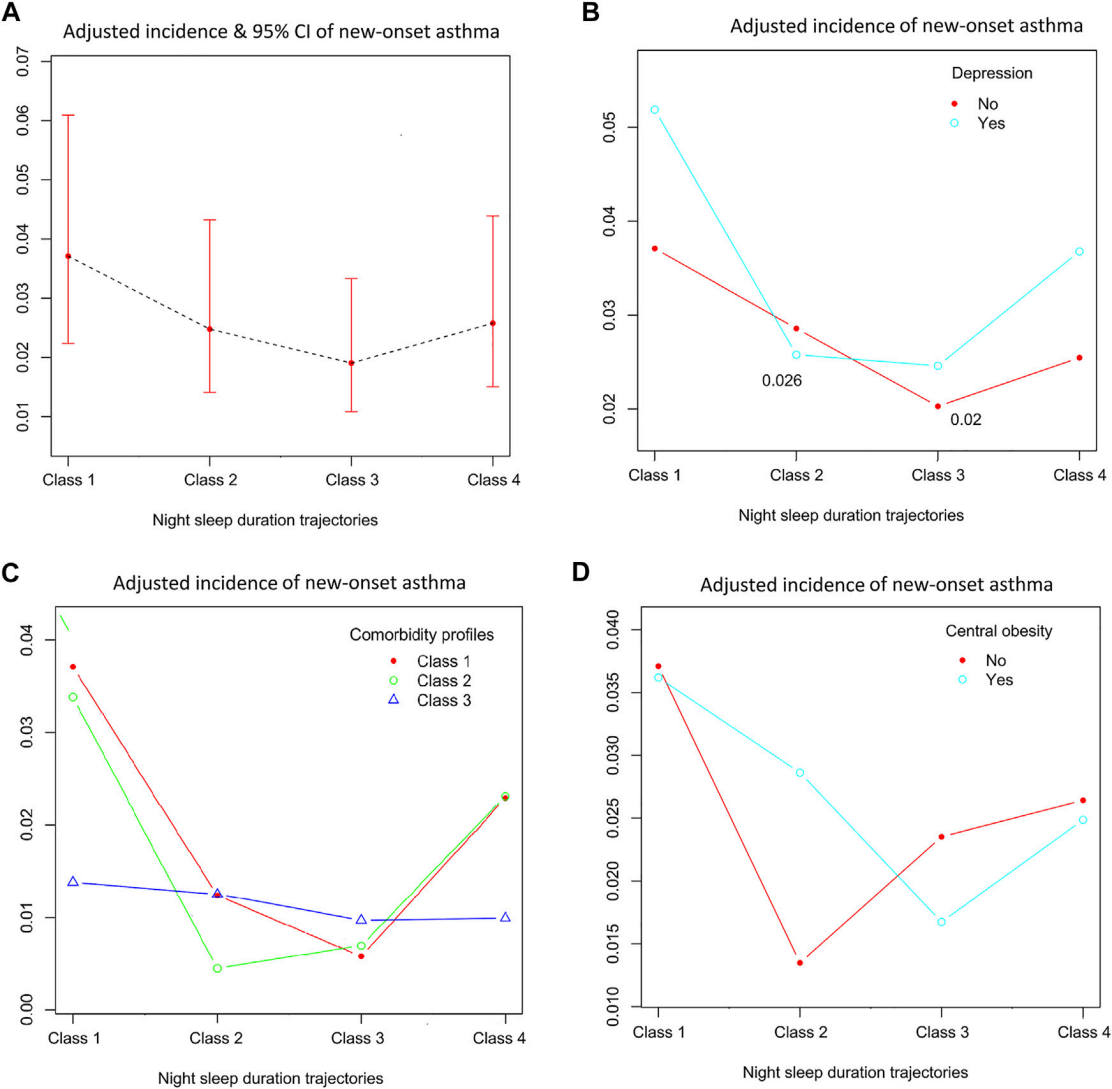
Associations between the incidence of new-onset asthma with night sleep duration in different study populations. Four night sleep duration trajectories: class 1, dominant short; class 2, dominant healthy-long; class 3, long decreasing; class 4, short increasing. **(A)** all study population; **(B)** study population stratified by depression; **(C)** study population stratified by three comorbidity profiles(class 1, dominant heart diseases or risks; class 2, multiple disorders; class 3, minimal or least disorders); **(D)** study population stratified by central obesity (China. 2022).

Table 2 showed the characteristics of the study population grouped by night sleep duration trajectories. The rank of incident asthma was dominant short (3.7%), short increasing (2.5%), dominant healthy-long (2.3%), and long decreasing (1.8%). Three models suggested that three other night sleep durations were associated with a significantly decreasing risk of developing asthma than the dominant short night sleep duration trajectory (see Table 3; Figure 3A). Napping duration trajectories had no significant effect correlation with incident asthma. After adjusting the potential confounding factors in model 3, long decreasing (adjusted OR = 0.51, 95% CI: 0.34–0.76) and short increasing (adjusted OR = 0.69, 95% CI: 0.48–0.98) trajectories and the dominant healthy-long trajectory (adjusted OR = 0.66, 95% CI: 0.49–0.998, *p <* 0.05) saw a significantly lower incidence of asthma than the dominant short night sleep duration trajectory.

**TABLE 2:** The characteristics of four night sleep duration trajectories in individuals without asthma from the China Health and Retirement Longitudinal Study.

N	Dominant short	Dominant healthy-long	Long decreasing	Short increasing	*p*
	2,480	1,405	1875	1895	
Total person years	17,230	9,783	13,064	13,202	
Sex					0.008
Male	1,134 (45.7%)	703 (50.0%)	915 (48.8%)	856 (45.2%)	
Female	1,346 (54.3%)	702 (50.0%)	960 (51.2%)	1,039 (54.8%)	
Age					0.086
<65 years	1837 (74.1%)	1,079 (76.8%)	1,441 (76.9%)	1,414 (74.6%)	
>65 years	643 (25.9%)	326 (23.2%)	434 (23.1%)	481 (25.4%)	
Region					0.006
North	653 (26.3%)	406 (28.9%)	555 (29.6%)	508 (26.8%)	
East and central	923 (37.2%)	553 (39.4%)	720 (38.4%)	769 (40.6%)	
West	904 (36.5%)	446 (31.7%)	600 (32.0%)	618 (32.6%)	
Urban/rural					0.043
Rural	1,504 (60.6%)	902 (64.2%)	1,197 (63.8%)	1,157 (61.1%)	
Urban	976 (39.4%)	503 (35.8%)	678 (36.2%)	738 (38.9%)	
Education levels					0.103
Low	1,111 (44.8%)	589 (41.9%)	782 (41.7%)	858 (45.3%)	
Middle	1,246 (50.2%)	745 (53.0%)	1,005 (53.6%)	962 (50.8%)	
High	123 (5.0%)	71 (5.1%)	88 (4.7%)	75 (4.0%)	
Married status					0.02
Unmarried	309 (12.5%)	134 (9.5%)	192 (10.2%)	201 (10.6%)	
Married	2,171 (87.5%)	1,271 (90.5%)	1,683 (89.8%)	1,694 (89.4%)	
Low skeletal strength				0.425	
No	1967 (79.3%)	1,127 (80.2%)	1,498 (79.9%)	1,541 (81.3%)	
Yes	513 (20.7%)	278 (19.8%)	377 (20.1%)	354 (18.7%)	
Smoking					0.04
Never	1,545 (62.3%)	822 (58.5%)	1,137 (60.6%)	1,185 (62.5%)	
Ever	216 (8.7%)	107 (7.6%)	151 (8.1%)	138 (7.3%)	
Current	719 (29.0%)	476 (33.9%)	587 (31.3%)	572 (30.2%)	
Alcohol					0.47
Never	1,696 (68.4%)	944 (67.2%)	1,268 (67.6%)	1,269 (67.0%)	
Ever	205 (8.3%)	127 (9.0%)	147 (7.8%)	141 (7.4%)	
Current	579 (23.3%)	334 (23.8%)	460 (24.5%)	485 (25.6%)	
Body mass index					0.316
Underweight	203 (8.2%)	89 (6.3%)	127 (6.8%)	127 (6.7%)	
Normal weight	1,271 (51.2%)	754 (53.7%)	978 (52.2%)	997 (52.6%)	
Overweight	710 (28.6%)	419 (29.8%)	546 (29.1%)	547 (28.9%)	
Obesity	296 (11.9%)	143 (10.2%)	224 (11.9%)	224 (11.8%)	
Central obesity					0.975
No	744 (30.0%)	419 (29.8%)	567 (30.2%)	578 (30.5%)	
Yes	1736 (70.0%)	986 (70.2%)	1,308 (69.8%)	1,317 (69.5%)	
Depression					<0.01
No	1,489 (60.0%)	1,044 (74.3%)	1,371 (73.1%)	1,170 (61.7%)	
Yes	991 (40.0%)	361 (25.7%)	504 (26.9%)	725 (38.3%)	
Comorbidity profiles				<0.01	
Class 1	264 (10.6%)	132 (9.4%)	180 (9.6%)	190 (10.0%)	
Class 2	295 (11.9%)	105 (7.5%)	139 (7.4%)	219 (11.6%)	
Class 3	1921 (77.5%)	1,168 (83.1%)	1,556 (83.0%)	1,486 (78.4%)	
Napping duration trajectories				<0.01	
Short increasing	1,256 (50.6%)	652 (46.4%)	867 (46.2%)	971 (51.2%)	
Stable normal	466 (18.8%)	246 (17.5%)	337 (18.0%)	330 (17.4%)	
Long decreasing	758 (30.6%)	507 (36.1%)	671 (35.8%)	594 (31.3%)	
New-onset asthma					<0.01
No	2,388 (96.3%)	1,373 (97.7%)	1842 (98.2%)	1847 (97.5%)	
Yes	92 (3.7%)	32 (2.3%)	33 (1.8%)	48 (2.5%)	

Comorbidity profiles: class 1, dominant heart diseases or risks; class 2, multiple disorders; class 3, minimal or least disorders.

**TABLE 3:** Associations between the incidence of new-onset asthma with night sleep duration and napping duration trajectories.

	Model 1	Model 2	Model 3
Night sleep duration			
Dominant short	Ref	Ref	Ref
Dominant healthy-long	0.62 (0.41, 0.93)^+^	0.61 (0.41, 0.93)^+^	0.66 (0.44, 0.998)^+^
Long decreasing	0.47 (0.32, 0.71)^$^	0.47 (0.32, 0.71)^$^	0.51 (0.34, 0.76)^$^
Short increasing	0.68 (0.48, 0.97)^+^	0.68 (0.48, 0.98)^+^	0.69 (0.48, 0.98)^+^
Napping duration			
Short increasing	Ref	Ref	Ref
Stable normal	1.05 (0.72, 1.53)	1.06 (0.73, 1.55)	1.03 (0.71, 1.51)
Long decreasing	0.86 (0.62, 1.20)	0.86 (0.62, 1.19)	0.84 (0.61, 1.17)

Model 1 adjusted the following variables: sex, age, region, urban/rural, education levels, and married status.

Model 2 adjusted the following variables: sex, age, region, urban/rural, education levels, married status, smoking, alcohol, body mass index, and low skeletal strength.

Model 3 adjusted the following variables: sex, age, region, urban/rural, education levels, married status, smoking, alcohol, body mass index, low skeletal strength, depression, central obesity, and comorbidity profiles.

^+^ *p* < 0.05; ^$^ *p* < 0.01.

Figure 3 demonstrated the effects of night sleep duration trajectories in incident asthma for all of the study population and specific populations. Generalized additive analysis with binomial regression provided an adjusted incidence of new-onset asthma for each specific population. The highest adjusted incidence (5.2%) of new-onset asthma was shown in individuals with depression and dominant short night sleep duration trajectory after adjusted model 3 plus napping duration trajectories. A minimal or least disorders profile was associated with a significant reduction of new-onset asthma than two other comorbidity profiles in the dominant short night sleep duration trajectory (see Figure 3C). However, the difference in incident asthma among four night sleep duration trajectories seemed to be insignificant in the minimal or least disorders profile.

Mediation analyses suggested that depression provided 5.4% (95% CI: 0.09%, 15.2%, *p* = 0.042) of the total effect in the association between dominant short night sleep duration trajectory and incident asthma. Central obesity (average mediated proportion = −0.01%, 95% CI: −1.17%, 1.27%, *p* = 0.948) and comorbidity profiles seemingly had no significant mediation effect.

## Discussion

To our knowledge, this is a new prospective study to investigate the relationship between incident asthma with different night sleep duration and napping duration trajectories in Chinese middle-aged and older adults. Our study had the following findings. Firstly, a dominant short night sleep duration trajectory was associated with an increased risk of developing asthma, especially in individuals with depression. Secondly, the incidence of new-onset asthma among different napping duration trajectories had no significant difference. Thirdly, a dominant short night sleep duration trajectory might affect the occurrence of asthma by mediating the effect of depression. Fourthly, people with dominant heart diseases or risks and multiple disorders profiles were more prone to incident asthma than someone with a minimal or least disorders profile.

In our study, latent profile class identified a dominant short night sleep duration trajectory during the 7-year follow-up period, which is more available to determine the cumulative impact of long-term exposure to short sleep duration on the risk of developing asthma than the single measurement of sleep duration in cross-sectional studies. We also found two other night sleep duration trajectories (long decreasing and short increasing) with short-term exposure to short sleep duration. Individuals in long decreasing and short increasing trajectories had a significantly lower risk of developing asthma compared with the dominant short night sleep duration trajectory. Our study indicated that the improvement of sleep duration potentially weakens the adverse effects that are secondary to short sleep duration. Inflammatory processes were regarded as an important mechanism linking short sleep duration and asthma. A persistent short sleep duration trajectory was associated with higher levels of systemic inflammation than a good sleep duration trajectory [25]. Studies suggested that depression and its severity may be attributed to short sleep duration [26, 27]. Our study also found that the prevalence of depression in the dominant short night sleep duration trajectory was higher than those in three other night sleep duration trajectories. We speculated that short sleep duration has the ability to affect the occurrence and development of asthma by increasing the risk of depression. Mediation analysis demonstrated that depression mediated up to 5.4% of the association between dominant short night sleep duration trajectory and incident asthma. Taken together, depression may be considered a potential pathway linking asthma and short sleep duration.

Two cross-sectional studies stratified weight status by BMI and then suggested that short sleep duration in overweight but not normal weight was accompanied by an increased risk of asthma compared with healthy sleep duration [8, 13]. This study demonstrated that a dominant short night sleep duration trajectory had a significantly higher incidence of asthma rate than a long decreasing trajectory in multiple specific populations (depression, central obesity, a dominant heart diseases or risks profile, and a multiple disorders profile) with the exception of a minimal or least disorders profile. In addition, a minimal or least disorders profile harbored a significantly lower risk of developing asthma than two other comorbidity profiles of the dominant short night sleep duration trajectory. Other studies have confirmed that short sleep duration seems to have a significant effect on clinical outcomes in individuals with some underlying diseases at baseline (such as hypertension, metabolic syndrome, cardio-metabolic risk factors, and possible vascular cognitive impairment) but not those without some underlying diseases [28–31]. The pathophysiological mechanism of this phenomenon remains unknown. A possible explanation is that obesity or depression or a multiple disorders profile is associated with a low-grade level of systemic inflammation. Short sleep duration may have additive effects on systemic inflammation on the basis of underlying diseases and then lead to a significant impact on clinical outcomes. In addition, a previous study indicated either common potential mechanisms, such as shared environmental or genetic exposures, or a possible causal association between asthma with short sleep duration and comorbidity profiles [7]. We conclude that our findings on the associations between night sleep duration trajectories with incident asthma in specific comorbidity profiles are novel and important. We hope that our study is a starting point for precision prevention and early intervention against new-onset asthma by way of screening and treating comorbidity profiles.

Children with asthma were associated with a higher frequent number of nap days than those without asthma [32], while the association of napping duration with incident asthma has not been studied. Long napping duration (≥60 min) was found to bring adverse clinical outcomes in cross-sectional studies [16, 17]. This prospective study suggested no significant effect of napping duration trajectories on incident asthma. Three napping duration trajectories had health napping duration after CHARLS wave 3, thus we did not assess the effect of continuously long napping duration on incident asthma. The association between napping duration and asthma remains to be further explored.

The main strength of our study exists in determining the associations between incident asthma with night sleep duration, napping duration, and comorbidities by using a national population-based prospective study and multiple statistical analyses. The major limitation is that the data on sleep, asthma, and comorbidity come from the questionnaire’s results, which has the potential for selection bias. A small proportion of people with asthma never seek help for clinicians, which potentially led to the underdiagnosis of incident asthma. However, a series of follow-up visits with CHARLS physical examinations may help individuals to improve their health consciousness and reduce the aforementioned selection bias. Additional limitations include the lack of other sleep disorders (such as insomnia and sleep apnea), which can potentially affect the final results. In addition, each individual is assigned based on the highest probability of belonging to one of the latent classes but not actually belonging to a single group in latent class analysis. Therefore, individuals may have a healthy sleep and napping duration at a certain time.

### Conclusion

In conclusion, this study found there is an association between a dominant short night sleep duration trajectory and an increasing incidence of new-onset asthma in the Chinese middle-aged and older population, especially individuals with depression. However, the incidence of asthma among four night sleep duration trajectories showed no significant difference in the minimal or least disorders profile. The findings indicate that precision prevention and early intervention may be targeted for high-risk groups to reduce the secondary burdens of asthma. Further studies are warranted to further corroborate our results by using objective measures of sleep duration, asthma, and comorbidities.

## Data Availability

The data underlying this study were obtained from the open China Health and Retirement Longitudinal Study database. All relevant data are within the paper and its Supplementary Material.
