# Supplementary material for: Associations Between Incident Asthma With Comorbidity Profiles, Night Sleep Duration, and Napping Duration Trajectories: A 7-Year Prospective Study
Source: Int J Public Health. 2022 Jul 1;67:1604939. doi: 10.3389/ijph.2022.1604939 (PMC9305997; doi:10.3389/ijph.2022.1604939)
Supplement: Supplementary file 1 [file DataSheet1.doc]

| Table S1: Fit indices for latent class analysis models with 2–8 classes in our study. (China 2022) | | | | | | |
| --- | --- | --- | --- | --- | --- | --- |
|  | AIC | BIC | aBIC | Entropy | LMRt | BLRt |
| Night sleep duration trajectories | | |  |  |  |  |
| Class 2 | 101863 | 101988 | 101931 | 0.727 | *P<0.01* | *P<0.01* |
| Class 3 | 99904 | 100064 | 99991 | 0.843 | *P<0.01* | *P<0.01* |
| Class 4 | 97132 | 97327 | 97238 | 0.899 | *P<0.01* | *P<0.05* |
| Class 5 | 99206 | 99435 | 99330 | 0.833 | *P<0.01* | *P<0.01* |
| Class 6 | 97754 | 98018 | 97897 | 0.933 | *P<0.01* | *P<0.01* |
| Class 7 | 99981 | 99280 | 99143 | 0.85 | *P<0.01* | *P<0.01* |
| Class 8 | 102071 | 102161 | 102119 | 0.835 | *P<0.01* | *P<0.01* |
| Napping duration trajectories | | |  |  |  |  |
| Class 2 | 87097 | 87187 | 87146 | 0.932 | *P<0.01* | *P<0.01* |
| Class 3 | 82388 | 82513 | 82456 | 0.996 | *P<0.01* | *P<0.01* |
| Class 4 | 83794 | 83954 | 83880 | 0.939 | *P<0.01* | *P<0.01* |
| Class 5 | 83353 | 83547 | 83458 | 0.932 | *P<0.01* | *P<0.01* |
| Class 6 | 82679 | 82909 | 82804 | 0.955 | *P<0.01* | *P<0.01* |
| Class 7 | 82510 | 82774 | 82653 | 0.902 | *P=1* | *P=1* |
| Class 8 | 82197 | 82495 | 82359 | 0.889 | *P=1* | *P=1* |
| Comorbidity profiles | |  |  |  |  |  |
| Class 2 | 52721 | 52908 | 52822 | 0.583 | *P<0.01* | *P<0.01* |
| Class 3 | 52110 | 52195 | 52164 | 0.632 | *P<0.01* | *P<0.01* |
| Class 4 | 52065 | 52446 | 52272 | 0.543 | *P<0.01* | *P<0.01* |
| Class 5 | 52024 | 52503 | 52284 | 0.548 | *P<0.05* | *P<0.01* |
| Class 6 | 52023 | 52599 | 52335 | 0.643 | *P=0.68* | *P=0.24* |
| Class 7 | 52026 | 52699 | 52391 | 0.593 | *P=0.56* | *P=0.67* |
| Class 8 | 52031 | 52802 | 52449 | 0.552 | *P=0.81* | *P=0.67* |
| Notes: AIC, Aikaike’s Information Criterion ; BIC, Bayesian Information Criterion; aBIC, Adjusted Bayesian Information Criterion; LMRt, Lo-Mendell-Rubin test; BLRt, Bootstrap Likelihood ratio test | | | | | | |


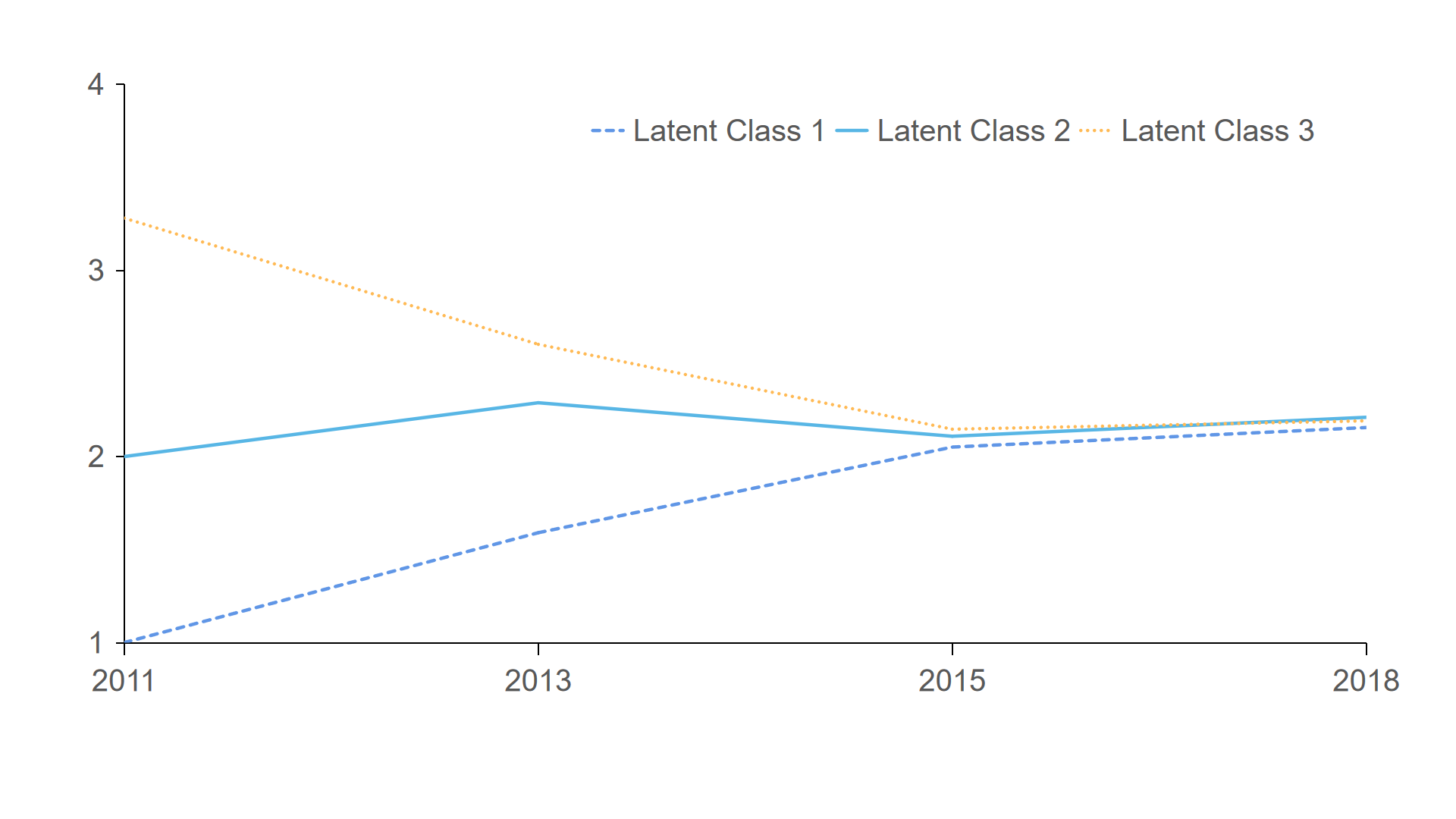


Figure S1：Latent class analyses of napping duration trajectories in individuals without asthma from the China Health and Retirement Longitudinal Study 2011: class 1, short increasing(reference, n=3746, 48.9%); class 2, stable normal (n=1379, 18%); class 3, long decreasing(n=2530, 33.1%).Napping duration was categorized as four groups: (1) 0 minutes as no nap; (2)0-59 minutes as health napping duration; (3)60-119 minutes as long napping duration; (4)≥120 minutes as very long napping duration. (China 2022)
